# *Citrus lemon* essential oil: chemical composition, antioxidant and antimicrobial activities with its preservative effect against *Listeria monocytogenes* inoculated in minced beef meat

**DOI:** 10.1186/s12944-017-0487-5

**Published:** 2017-08-03

**Authors:** Anis Ben Hsouna, Nihed Ben Halima, Slim Smaoui, Naceur Hamdi

**Affiliations:** 1 Department of Life Sciences, Faculty of Sciences of Gafsa, Zarroug, 2112 Gafsa, Tunisia; 2 Biotechnology and Plant Improvement Laboratory, Centre of Biotechnology of Sfax, PO Box 1177, Road Sidi Mansour 6 km, 3018 Sfax, Tunisia; 3 Laboratory of Microorganisms and Biomolecules of the Center of Biotechnolgy of Sfax-Tunisia, Road of Sidi mansour, Km 6 B.P. 1117, 3018 Sfax, Tunisia; 4 College of Science and Arts, Qassim University, P.O. Box 53, Al-Rass, Saudi Arabia; 5 High Institute of Environmental Science and Technologies (HIEST), Borj Cedria, Tunisia

**Keywords:** *Citrus limon*, Essential oil, Antioxidant activity, Antimicrobial activity, *Listeria monocytogenes*, minced beef meat

## Abstract

**Background:**

Lemon (*Citrus limon*) is a flowing plant belonging to the *Rutaceae* family. *Citrus* plants constitute one of the main valuable sources of essential oil used in foods and medicinal purposes.

**Methods:**

In this study, we assessed chemical composition, antioxidant and antimicrobial activities of *C. limon* essential oil (*Cl*EO) with its preservative effect against *Listeria monocytogenes* inoculated in minced beef meat. Gas chromatography/mass spectrometry (GC-MS) was used to identify the major components of the obtained *Cl*EO. The antioxidant activities of this *Cl*EO were determined according to the β-carotene bleaching assay, as well as by 2.2-diphenyl-1-picrylhydrazyl (DPPH) radical scavenging activity. For antimicrobial activity, agar well diffusion method was used and the minimum inhibitory concentrations (MICs) as well as the minimum fungicidal concentrations (MFCs) were determined. The in situ effect of the *Cl*EO was evaluated through physicochemical parameters (pH and thiobarbituric acid reactive substances (TBARS), as well as against *L. monocytogenes* in minced beef meat model.

**Results:**

Twenty one components were identified in the *Cl*EO and the two dominant compounds were limonene (39.74%) and β-Pinene (25.44%). This *Cl*EO displayed an excellent DPPH scavenging ability with an extract concentration providing 50% inhibition (IC_50_) of 15.056 μg/ml and a strong β-carotene bleaching inhibition after 120 min of incubation with an IC_50_ of 40.147 μg/ml. The MICs varied from 0.039 to 1.25 mg/ml for Gram positive bacteria and from 0.25 to 2.5 mg/ml for Gram-negative bacteria. The meat preserving potential of *Cl*EO was investigated against *L. monocytogenes*. *Cl*EO successfully inhibited development of *L. monocytogenes* in minced beef meat. The application of *Cl*EO at a 0.06 and 0.312 mg/g, may open new promising opportunities for the prevention of contamination from and growth of pathogenic bacteria, particularly *L. monocytogenes*, during minced beef meat storage at 4 °C. Additionally, during storage period, physicochemical values (pH and TBARS) were higher in control meat than treated meat with *Cl*EO suggesting an efficient antioxidant activity of *Cl*EO.

**Conclusion:**

It was suggested that the *Cl*EO may be a new potential source as natural antimicrobial and antioxidant agents applied in food systems and pharmaceutical industry.

## Background

*Citrus* are the most important crops in the world in terms of production according to the Food and Agricultural Organisation (FAO), with 240,780 million metric tons produced in 2013 [1]. *Citrus* plants are grown in many countries all over the world and among the major African citrus-producing countries is Tunisia. Thus, *Citrus* would be considered as one of the most economically important crops in Tunisia. The genus *Citrus* belongs to the *Rutaceae* family that comprises of about 140 genera and 1300 species and, for instance, *Citrus limon* (Lemon) is among important species of genus *Citrus* [2]. Essential oils were composed of many valuable natural products that may be described as mixtures of hydrocarbons, oxygenated compounds and nonvolatile residues. They include terpenes, sesquiterpenes, aldehydes, alcohols, esters and sterols [3]. *Citrus* plants constitute one of the main sources of essential oil, which are extensively studied for their potential uses in the food industry [4].

Foods contaminated with *Listeria monocytogenes, Staphylococcus aureus, Escherichia coli* O157:H7 and *Salmonella* has been reported as the causal agents of foodborne diseases [5, 6]. One of the most important psychrotrophic food pathogens related to anaerobically packed cooked meat products and shelf-life failures of conserved foods is *Listeria monocytogenes*. This organism is the causal agent of listeriosis, a disease that can be serious and is often fatal in susceptible individuals, caused by eating contaminated food [7]. Thus, to prevent contamination during the production, sale and distribution and to extend the shelf life time of raw and/or processed foods, synthetic additives should be used. However, there is a strong debate about the safety aspects of these chemical preservatives since they are considered responsible for many carcinogenic and teratogenic attributes as well as residual toxicity [8]. Thus, a growing attention is being paid to plants and herbs naturally derived compounds as a new alternative to prevent the proliferation of microorganism and protect food from oxidation. Generally, little information exists on the in vivo antimicrobial efficacy of plant essential oils against food-borne pathogens in meat. To the best of our knowledge, the antimicrobial activity of *Citrus limon* essential oil (*Cl*EO) against a wide range of food-associated microorganisms (bacteria, moulds, and yeasts) has not been studied. The purposes of the present work were (i) to evaluate the chemical composition of Tunisian lemon EO (*Cl*EO) by GC-MS, (ii) to study in vitro the antioxidant and antimicrobial activities of *Cl*EO, (iii) to assess the effect of *Cl*EO on physicochemical of raw minced beef meat stored at 4 °C, and (iv) to determine the efficacy of *Cl*EO in inhibiting *L. monocytogenes* growth in raw minced beef meat during refrigerated storage.

## Methods

### Chemicals

All chemicals used were of analytical reagent grade. All reagents were purchased from Sigma-Aldrich-Fluka (Saint-Quentin France).

### Collection of plant material

The flowers of *Citrus limon* were collected by hand in the beginning of Avril 2015 from the Cap Bon of Tunisia, precisely in the surroundings of Nabeul localized in 5 m of altitude and 1044 of longitude. In Mars 2015, Nabeul average rainfall was 31 mm, the average of Temperature was 20.5 °C and humidity was 79% (NIM-National Institute of Meteorology-Tunisia, 2015). Then the botanical identification of *Citrus lemon* was conducted by Professor Ferjeni Ben Abduallah, botanist in the Faculty of Science of Sfax, Tunisia.

### Essential oil extraction

The oil extraction was obtained from 1 kg fresh plant by steam distillation during 3 h using a Clevenger-type apparatus [9]. The aqueous phase was extracted with dichloromethane (3 × 50 ml) and dried with anhydrous sodium sulphate (PubChem CID: 24,243). After filtration the solvent is eliminated by pressure distillation reduced in rotary evaporator and pure oil was stored at 4 °C in obscurity till the beginning of *Cl*EO analysis. The amount of oil obtained from each plant material was calculated as:

Oil (%*v*/*w*) = observed volume of oil (ml)/weight of sample (g) × 100.

The *Cl*EO was solubilised in *n*-Hexane (PubChem CID: 8058) for gas chromatography and mass spectrometry analysis.

### Antioxidant testing assays

#### DPPH radical scavenging activity

Radical scavenging activity of the different fractions was determined using DPPH radical as a reagent according to the method of Kirby and Schmidt with some modifications [10]. Briefly, 1 ml of a 4% (*w*/*v*) solution of DPPH radical in ethanol (PubChem CID: 702) was mixed with 500 μl of sample solutions (different concentrations). The mixture was incubated for 20 min in the dark at room temperature. Scavenging capacity was read spectrophotometrically by monitoring the decrease of the absorbance at 517 nm. Lower absorbance of the reaction mixture indicates higher free radical scavenging activity. Ascorbic acid was used as standard. DPPH radical scavenging activity (%) was calculated according to the formula: DPPH radical scavenging activity (%) = [(OD_blank_ − OD_sample_)/OD_blank_] × 100.

OD_blank_ is the absorbance of the control reaction containing all reagents except the tested compound. OD_sample_ is the absorbance of the test compound. Extract concentration providing 50% inhibition (IC_50_) was calculated from the graph plotting inhibition percentage against extract concentration. Tests were carried out in triplicate.

#### β-Carotene bleaching assay

The antioxidant activity was determined according to the β-carotene bleaching method described by Pratt [11]. A stock solution of β-carotene/linoleic acid mixture was prepared as follows: 0.5 mg of β-carotene (PubChem CID: 5,280,489) was dissolved in 1 ml of chloroform (PubChem CID: 6212) with 25 μl of linoleic acid (PubChem CID: 5,280,450) and 200 mg of Tween-20 (PubChem CID: 443,314). Chloroform was completely evaporated, using a vacuum evaporator. Then, 100 ml of distilled water, saturated with oxygen (30 min), were added and the obtained solution was vigorously shaken. Four ml of this reaction mixture were dispensed into test tubes and 200 μl of each sample, prepared at different concentrations, were added. The emulsion system was incubated for 2 h at 50 °C. The same procedure was repeated with Butylated hydoxytoluene (BHT) as positive control and a blank as a negative control. After this incubation period, the absorbance of each mixture was measured at 490 nm. Antioxidant activity in β-carotene bleaching model in percentage (A %) was calculated with the following equation: A %  = 1 − (A_0_ – A_t_/A^’^_0_−A^’^_t_) × 100, where A_0_ and A’_0_ are absorbances of the sample and the blank, respectively, measured at zero time, and A _t_ and A’_t_ are absorbances of the sample and the blank, respectively, measured after 2 h. All tests were carried out in triplicate.

### Antimicrobial activity

#### Microorganisms and growth conditions

Authentic pure cultures of bacteria and fungi were obtained from international culture collections: American type culture collection (ATCC) and the local culture collection of the Center of Biotechnology of Sfax, Tunisia. They included Gram-positive bacteria: *Bacillus subtilis* ATCC 6633*, Bacillus cereus* ATCC 14579*, Staphylococcus aureus* ATCC 25923*, Staphylococcus epidermidis* ATCC 12228*, Enterococcus faecalis* ATCC 29212 and *Listeria monocytogenes* ATCC 19117 and Gram-negative bacteria: *Salmonella enterica* ATCC 43972*, Escherichia coli* ATCC 25922 and *Pseudomonas aeruginosa* ATCC 9027.

The following fungal strains were also tested: *Aspergillus niger* CTM 10099*, Aspergillus flavus* (food isolate)*, Aspergillus nidulans* (food isolate), *Aspergillus fumigatus* (food isolate), *Fusarium graminearum* (ISPAVE 271*), Fusarium oxysporum* (CTM10402)*, Fusarium culmorum* (ISPAVE 21w) and *Alternaria alternata* (CTM 10230)*.* The bacterial strains were grown on Mueller Hinton broth (Bio-Rad, France) at 37 °C for 12–14 h while potato dextrose agar (PDA) (1.5% agar) at 28 °C for 4 days were used for fungi. Inocula were prepared from an overnight broth culture by their dilution in saline solution to approximately 10^7^ colony-forming units (CFU)/ml for bacteria and 10^5^ spores/ml for fungus.

### Agar diffusion method

Antibacterial and antifungal tests were performed by agar well diffusion method as described by Tagg and McGiven [12] and broth microdilution assay using sterile Mueller–Hinton media (Bio-Rad, France) for bacterial strains and potato dextrose agar (Bio-Rad,France) for antifungal tests. Fifteen milliliters of the molten agar (45 °C) were poured into sterile petri dishes (Ø 90 mm). Working cell suspensions were prepared and 100 μl were evenly spread onto the surface of the agar plates of Mueller-Hinton agar (Oxoid Ltd., UK) for bacteria, or potatoes dextrose agar medium (Oxoid Ltd., UK) for fungi. Once the plates had been aseptically dried, 06 mm wells were punched into the agar with a sterile Pasteur pipette. The *Cl*EO were dissolved in dimethylsulfoxide (DMSO)/water (1/1) and sterile water to a final concentration of 50 mg/ml. Thus, 50 μl were placed into the wells and the plates were incubated at 37 °C for 24 h for bacterial strains and 72 h for fungi at 28 °C. Gentamicin (10 μg/wells), Amphotericin B (PubChem CID: 5,280,965) at 20 μg/wells and DMSO served as positive and negative control. Antimicrobial activity was evaluated by measuring the diameter of circular inhibition zones around the well. Tests were performed in triplicate.

### Determination of MIC and MFC

Minimum inhibitory concentrations (MICs) of *Cl*EO were determined according to Gulluce et al. [13] against a panel of 21 microorganisms representing different species of different ecosystems. The test was performed in sterile 96-well microplates with a final volume in each microplate well of 100 μl. A stock solution of the *Cl*EO (50 mg/ml) was prepared in DMSO/water (1/9). The inhibitory activity of the *Cl*EO was properly prepared and transferred to each well in order to obtain a twofold serial dilution of the original sample and to produce the concentration range of 0.039–10 mg/ml. To each test well 10 μl of cell suspension were added to final inoculums concentrations of 10^6^ CFU/ml for bacteria and 10^5^ spores/ml for fungus. Positive growth control wells consisted of bacteria or fungi only in their adequate medium. DMSO/water (1/9) (PubChem CID: 679) was used as negative control. The plates were then covered with the sterile plate covers and incubated at 37 °C for 24 h for bacterial strains and 72 h for fungi at 28 °C. The MIC was defined as the lowest concentration of the total essential oil at which the microorganism does not demonstrate visible growth after incubation. As an indicator of microorganism growth, 25 μl of Thiazolyl Blue Tetrazolium Bromide (PubChem CID: 64,965) (MTT), indicator solution (0.5 mg/ml) dissolved in sterile water were added to the wells and incubated at 37 °C for 30 min. The colourless tetrazolium salt acts as an electron acceptor and is reduced to a red-coloured formazan product by biologically active organisms. Where microbial growth was inhibited, the solution in the well remained clear after incubation with MTT. The minimum fungicidal concentrations (MFCs) were determined by serial subcultivation of 10 μl in Potatoes Dextrose Agar (PDA) plates and incubated for 72 h at 28 °C. The lowest concentration with no visible growth was defined as the MFC, indicating ≥99.5% killing of the original inoculum. DMSO and ethanol were used as a negative control. The determinations of MIC and MFC values were done in triplicate.

### In situ effect of *Cl*EO

The in situ efficacy of the *Cl*EO was evaluated against *L. monocytogenes* in minced beef meat model according to the procedure described by Ben Hsouna et al. [5] and Careaga et al. [14] with slight modifications. Briefly, a fresh working culture of *L. monocytogenes* containing about 10^6^ CFU/ml was prepared by suspending 3–5 isolated colonies in 10 ml of Mueller-Hinton broth (MH) and was cultured overnight at 37 °C for 24 h to reach the stationary phase. Freshly post-rigor lean beef muscles were obtained from a slaughterhouse in Sfax-Tunisia and transported to the laboratory in insulated polystyrene boxes on ice within 1 h of the chopping process. A healthy and freshly slaughtered animal has its muscle sterile [15]. In order to reduce the number of microorganisms attached to the surface of beef muscle, each piece was immersed in boiling water for 5 min. The cooked surface of the muscle was eliminated with sterile knives under aseptic conditions. The pieces of meat prepared as described above were minced in a sterile grinder, and portions of 25 ± 0.1 g were placed in bags. Half of the meat samples were inoculated with 2 × 10^2^ CFU of *L. monocytogenes*/g of meat and mixed homogeneously for 3 min at room temperature to ensure proper distribution of the pathogen. Before inoculation of the second half of meat, the *Cl*EO was dissolved in 10% DMSO, filtered through 0.22 μm pore-size black polycarbonate filters (Millipore) and was then added two concentrations of 2MIC and 3MIC corresponding to 0.06 and 0.312 mg of *Cl*EO/g of meat, respectively; and mixed to evenly distribute the microorganisms. All bags containing the samples of meat were stored at 7 °C and examined at 0, 2, 4, 6, 8 and 10 days of storage for *L. monocytogenes* enumeration. After predetermined intervals, the *L. monocytogenes* isolation was done by removing the pieces aseptically and blending with 250 ml of MH. The samples were homogenized for 1 min, and incubated at 37 °C for 6 h. From this pre-enrichment (for the resuscitation of possible injured living cells), the remaining *L. monocytogenes* was determined by the plate colony count technique. After serial 10-fold dilution technique with physiological saline solution, 100 μl of each sample were spread onto surfaces of the Muller Hinton agar medium followed by incubation at 37 °C for 24 h. Sterile saline water was added in the untreated control instead of *Cl*EO, inoculated with the test bacteria and stored under the same conditions as the other samples. Three individual replicates of each experiment were performed, in all cases.

### Sample preparation

Raw meat beef was provided by a local supplier (slaughterhouse of Sfax, Tunisia). They were placed in insulated polystyrene boxes on ice and transferred to the laboratory within 1 h of slaughtering. Raw meat beef was raw minced by grinding in a sterile grinder. Concerning the conditioning of raw minced beef for storage at 4 °C studies, five equal portions (C, T1- T4): C (Control), T_1_ (*Cl*EO: 0.06%), T_2_ (*Cl*EO: 0.312%), T_3_ (BHT at 0.01%), T_4_ (*Cl*EO 0.312% and BHT at 0.01%). For each experiment, all runs were applied to a single batch of raw minced beef meat. Each formulation produced one mixture homogeneous which was vacuum stuffed into plastic casings to produce three ‘replicates’.

Meat samples were stored at 4 °C for 10 days and analyzed for pH and TBARS.

### pH determination

pH was determined for the homogeneous mixtures of meat with distilled water using a proportion of 1:10, *w*/*v* [16]. A 5 g portion of the sample was homogenized in 50 ml of distilled water (pH 7.00) and the mixture was filtered. The pH of the filtrate was measured using pH-meter (pH 210 Microprocessor pH Meter, HANNA instruments, Germany) at each sampling point.

### Thiobarbituric acid reactive substances (TBARS) value

TBARS were determined according to the method of Eymard et al. [17]. Two grams of sample were mixed with 100 μl of BHT (PubChem CID: 31,404) in ethanol (1 g/l) and 16 ml of trichloroacetic acid (TCA 50 g/l) (PubChem CID: 6421). Samples were homogenized by a kitchen blender for 10 min then filtered. Two ml of filtrate (or 2 ml of TCA for blank) were added to 2 ml of thiobarbituric acid (PubChem CID: 2,723,628) solution (20 mol/l). The tightly closed tubes were heated at 70 °C for 30 min and rapidly cooled in ice. Absorbance was read against the blank at 508 (A508nm), 532 (A532nm) and 600 (A600nm) with a spectrophotometer (T 60UV- visible Spectrophotometer, PG instruments). The absorbance measured at the maximum (A532 nm) was corrected for the baseline drift as follows: A532 nm corrected = A532 nm − [((A508 nm − A600 nm) × (600 – 532))/(600/508)] − A600 nm. Results were expressed as mg of malonaldehyde (MDA) equivalents per kg of sample (mg/kg) using the molar extinction coefficient of the MDA–TBA adduct at 532 nm (1.56 × 105 M − 1 cm − 1) according to Buege and Aust [18]. The MDA was determined using the following formula: mgMDAeq/kg = (A corrected × VTCA × 2 × MMDA . 10 – 2)/(1.56 × m).

### Gas chromatography–mass spectrometry (GC–MS)

The analysis of the essential oil was performed on a GC–MS HP model 6980 inert MSD (Agilent Technologies, J&W Scientific Products, Palo Alto, CA, USA), equipped with an Agilent Technologies capillary HP-5MS column (60 m length; 0.25 mm i.d.; 0.25 mm film thickness), and coupled to a mass selective detector (MSD5973, ionization voltage 70 eV; all Agilent, Santa Clara, CA). The carrier gas was Helium and was used at 1.2 ml/min flow rate. The oven temperature program was as follows: 1 min at 100 °C ramped from 100 to 280 °C at 5 °C min^−1^ and 25 min at 280 °C. The chromatograph was equipped with a split/split less injector used in the split less mode. Relative proportion of each component was expressed as percentage obtained by peak area normalization, all relative response factors being taken as one. Their Kovats indices were calculated using a homologous series of C_10_-C_22_ *n*-alkanes injected at the same conditions. Identification of components was assigned by matching their mass spectra with Wiley Registry of Mass Spectral Data 7th edition (Agilent Technologies, Inc.) and National Institute of Standards and Technology 05 MS (NIST) library data. Identifications were also made by comparison of their Kovats retention indices with private reference libraries and from the literature.

### Statistical analysis

A one-way analysis of variance (ANOVA) and Turkey’s post hoc test were performed to determine significant differences between the treatments using SPSS 19 statistical package (SPSS Ltd., Woking, UK). Microbiological data were transformed into logarithms of the number of colony forming units (CFU/g) and subjected to analysis of variance. Means and standard errors were calculated. Differences among the mean values of the various treatments were determined by the least significant difference test. A probability level of *P* < 0.05 was used in testing the statistical significance of all experimental data. Mean differences were separated by the least significance difference (LSD) procedure.

## Results and discussion

### Chemical composition of the essential oil

The essential oil obtained from the fresh flowers of *C. limon* by hydrodistillation had a light yellow colour. The oil yield was 3% (volume/fresh weight). The *Cl*EO was analyzed by GC-MS. Twenty one different components were identified in the EO of *Citrus limon*. The identified components, with their relative percentages and the retention time, are given in Table 1. The analyses revealed a complex mixture of the *Cl*EO consisting mainly of hydrocarbon monoterpene, oxygenated monoterpene and nitrogen compounds. Nine major detected components were found to be: β-Pinene (25.44%), Limonene (39.74%), Linalool (2.16%), α-Terpineol (7.30%), linalyl acetate(3.01%), Acétate geranyl (3.03%), Nerolidol (6.91%), Acetate neryl (1.74%) and Farnesol (4.28%). The minor components (< 1%) were identified as: α-Pinene (0.46%), Sabinene (0.44%), Myrcene (0.36%), 1,8-cineol (0.54%), cis-linalool oxide (0.53%) and Geranial (0.43%). In our experiment, the *Cl*EO isolated from Tunisian lemon samples contains the above-mentioned four predominant ingredients with different percentage. On the other hand, Ahmad et al. [19] reported that also limonene was the major chemical constituent in Indian oil. However, our results were different percentage from the reported one. From our experiment we observed that the percentage of chemical composition and predominant ingredients was not constant. The percentage of active ingredients in the essential oil from the natural samples is depends on geographical distribution as well as the environmental conditions such as temperature, rainfall, altitude, hours of sunshine, etc. [20]. On the other hand, the activities such as biological and chemical activities are always depends on the active ingredients in the oil [21]. The active ingredient such as limonene, α-pinene, β-pinene and β-myrcene are used in perfumes industry, flavorings and medicines as a local antiseptic and anesthetic [22].

**Table 1:** Chemical composition of essential oil isolated by hydrodistillation from flowers of *Cl*EO

No.	Rt (min)^b^	KI^c^	^a^Components	^d^%
1	7.83	939	α-Pinene	0.46
2	8.62	975	Sabinene	0.44
3	8.78	980	β-Pinene	25.44
4	8.82	991	Myrcene	0.36
5	9.87	1030	Limonene	39.74
6	9.98	1033	1,8-cineol	0.54
7	10.91	1074	cis-linalool oxide	0.53
8	11.30	1075	trans-linalool oxide	0.49
9	11.51	1078	Linalool	2.16
10	13.76	1178	Terpinen-4-ol	0.26
11	13.80	1180	Myrtenal	0.43
12	14.08	1189	α-Terpineol	7.30
13	14.91	1228	Nerol	0.99
14	15.59	12.57	linalyl acetate	3.01
15	16.05	1270	Geranial	0.43
16	16.82	1288	Indol	0.45
17	18.13	1337	anthraniate méthyl	0.66
18	18.41	1347	Acetate neryl	1.74
19	18.90	1383	Acétate geranyl	3.03
20	23.49	1534	Nerolidol	6.91
21	27.09	1705	Farnesol	4.28
			Monoterpene hydrocarbon	67.86
			Oxygenated monoterpenes	30.68
			Nitrogen components	1.11
			Total %	99.65

^a^Identification of components based on GC-MS Wiley 7.0 version library and National Institute of Standards and Technology 05 MS (NIST) library data

^*b*^ *Rt* retention time

^c^ *KI* Kovats Indices on HP-5MS Capillary Column in reference to C_10_-C_22_ n-alkanes injected in the same conditions

^d^Percentages are the means of two runs and were obtained from electronic integration measurements using a selective mass detector

### Antioxidant activities

**Fig. 1:**
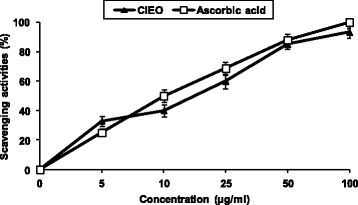
Scavenger effect of *Cl*EO at different concentrations 5, 10, 25, 50 and 100 μg/ml, on the stable 1,1-diphenyl-2-picrylhydrazyl radical (DPPH). Results are expressed as percentage decrement of absorbance at 517 nm with respect to control. Ascorbic acid was used as a positive control. Each value represents the mean ± SD of three experiments

Plants with radical scavenging property and antioxidant capacity are useful for medicinal applications and as food additive. So, in the present study, the antioxidant capacity of *Cl*EO was evaluated using DPPH radical scavenging method by comparing with the activity of the Ascorbic acid as a known antioxidant. The DPPH test aims at measuring the capacity of the essential oil to scavenge the stable free radical DPPH^.^ by donation of hydrogen atom or an electron [23]. If the extracts have the capacity to scavenge the DPPH free radical, the initial blue/purple solution will change to a yellow colour due to the formation of diphenylpicrylhydrazine. The effect of the different *Cl*EO on DPPH radical scavenging was compared to those of ascorbic acid, used as positive control, and appreciated by the determination of the IC_50_ values. As shown in Fig. 1, DPPH test revealed that the free radical-scavenging activity was a dose dependent manner. From the analysis of Fig. 1, we can conclude that the radical-scavenging activity of the *Cl*EO and positive controls ascorbic acid increased with increasing concentration (*P* <0.05). The current study demonstrated that *Cl*EO exhibited a strong radical scavenging activity as compared to the standard ascorbic acid.

**Fig. 2:**
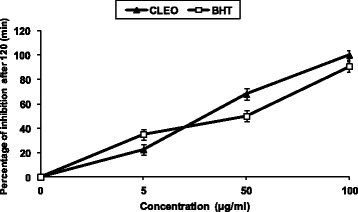
Antioxidant activities of *Cl*EO at different concentrations, 5, 50 and 100 μg/ml measured by β-carotene bleaching method. BHT was used as a positive control. Values are means ± SD (*n* = 3)

The inhibitory effect of the different *Cl*EO concentrations on lipid peroxidation was determined by the β-carotene/linoleic acid bleaching test. Figure 2 showed a various degree of the linoleic acid oxidation and subsequently the β-carotene bleaching after addition of the *Cl*EO and the BHT used as positive control at different concentrations. This antioxidant activity of the *Cl*EO was increased with the increasing concentration (*P*<0.05). Overall results were better than those provided by the radical-scavenging activity (Figs. 1 and 2). In fact, *Cl*EO displayed an excellent DPPH scavenging ability with an EC_50_ of 15.056 μg/ml and a strong β-carotene bleaching inhibition after 120 min of incubation with an EC_50_ of 40.147 μg/ml (Figs. 1 and 2). The inhibition of lipid peroxidation by addition of *Cl*EO could be used to improve the quality and stability of food products. *Cl*EO was able to quenching peroxide radicals and to terminate the peroxidation chain reaction.

The antioxidant activity may be ascribed to the presence of the same chemical components. Monoterpenes found in this essential oil may act as radical scavenging agents. It seems to be a general trend that the essential oils which contain monoterpene hydrocarbons, oxygenated monoterpenes and/or sesquiterpenes have greater antioxidative properties [24–26]. These activities may be attributed to the presence of 1,8-cineol, α-pinene, β-pinene and limonene found in *C. limon* essential oils. Essential oils are quite complex mixtures constituted by several tens of components, and this complexity makes it often difficult to explain the activity pattern. For this reason, many reports on the antioxidant potentials of the essential oils often refer to concepts such as synergism, antagonism and additivity.

Monoterpene hydrocarbons; particularly terpinolene, α- and γ-terpinene, could also be taken into account for the antioxidative activity observed, but obviously, none has stronger than that of oxygenated monoterpenes. The presence of strongly activated methylene groups in these molecules is probably the reason for this behavior. On the other hand, sesquiterpenes hydrocarbons and their oxygenated derivatives have very low antioxidant activity [27]. The findings above showed the presence of natural antioxidant property in *Cl*EO, which are better than that of BHT, a very efficient synthetic antioxidant agent which is widely used in food technology. Basically, interest has increased noticeably in the research of naturally occurring antioxidants for use in foods or medicinal materials as an alternative to synthetic antioxidants, which are being limited because of their possible toxicity [28, 29].

### Antimicrobial activities

The antibacterial activity of *C. limon* essential oil was evaluated against Gram + positive (*B. cereus, E. faecalis, S. aureus, S. epidermis, B. subtilis, L. monocytogenes* and *M. luteus*) and Gram-negative (*P. aeruginosa, E.coli, S. enteritidis* and *K. pneumoniae*) bacteria. The antibacterial activity was assessed by evaluating the inhibition zone (IZ) and the determination of MIC values. As can be seen in Table 2, *Cl*EO showed varying degrees of antibacterial activity against all strains tested. The inhibition zones were in the range of 13–26 mm. Among Gram positive bacteria, highest inhibitory zone was observed against *L. monocytogenes* (26 mm) followed by *B. cereus* (24 mm) and *S. aureus* (22 mm). Among Gram negative, highest inhibitory zone was observed against *S. enteritidis* (18 mm). The inhibition zone for Gentamicin (10 μg/well), which was used as positive controls for bacteria, ranged from 12 to 25 mm. Negative control did not show any inhibitory effect against the tested bacteria.

**Table 2:** Antibacterial activity of *Cl*EO against foodborne, spoiling bacteria and determination of the Minimum Inhibitory Concentrations (MICs) expressed in mg/ml

Strains	Inhibition zones diameter (mm)^a^		MIC (mg/ml)
	EO^b^	Gentamicin^c^	
Bacterial strains			
Gram positive			
*Bacillus subtilis* ATCC 6633	19 ± 0.8^a^	20 ± 0.2^a^	0.625 ± 0.4
*Bacillus cereus* ATCC 14579	24 ± 0.4^b^	20 ± 0.4^a^	1.25 ± 0.6
*Staphylococcus aureus* ATCC 25923	22 ± 0.6^b^	25 ± 0.8^b^	0.078 ± 0.5
*Staphylococcus epidermis* ATCC 12228	16 ± 0.8^a^	20 ± 0.5^b^	1.25 ± 0.6
*Enterococcus faecalis* ATCC 29212	15 ± 0.4^b^	12 ± 0.2^a^	0.625 ± 0.5
*Listeria monocytogenes* ATCC 19117	26 ± 0.3^b^	15 ± 0.0^a^	0.039 ± 0.3
Gram negative			
*Salmonella enterica* ATCC 43972	18 ± 0.9^a^	18 ± 0.8^a^	0.625 ± 0.4
*Escherichia coli* ATCC 25922	15 ± 0.5^a^	21 ± 1.0^b^	1.25 ± 0.6
*Pseudomonas aeruginosa* ATCC 9027	14 ± 0.3^a^	18 ± 0.7^b^	2.5 ± 0.4

Values are given as mean ± S.D. of triplicate experiments

^a^Diameter of inhibition zones of including diameter of disc 6 mm

^b^ *EO Lemon citrus* essential oil (50 μl/well)

^c^The used concentration of Gentamicin was 10 μg/well

Most studies investigating the action of essential oils against food spoilage organisms and food borne pathogens agree that, generally, essential oils are slightly more active against Gram-positive than Gram-negative bacteria [30–33]. That Gram-negative organisms are less susceptible to the action of antibacterials is perhaps to be expected, since they possess an outer membrane surrounding the cell wall, which restricts diffusion of hydrophobic compounds through its lipopolysaccharide covering. However, our results show that Gram-positive bacteria are more sensitive to the investigated oil, with a range of 0.039 to 1.25 mg/ml than Gram-negative bacteria in the range of 0.625 to 2.5 mg/ml, which has been confirmed and extended in the present studies. From a comparison of our results with values reported in the literature, it is interesting the oil showed an antimicrobial effect in the concentration range as the most active essential oils [34, 35]. The antimicrobial activity of essential oils is believed to be associated with phytochemical components. Essential oils are volatile and odorous principles of plant secondary metabolism which have wide applications in food flavoring and preservation industries [36]. Recently, some researchers have reported that monoterpene or sesquiterpene hydrocarbons and their oxygenated derivatives, which are the major components of essential oils, exhibit potential antimicrobial activities [37]. These findings strongly supported the results of this study as the essential oil from *C. limon* s was also found to contain these components, which confirmed its efficacy as natural antimicrobial agent.

The antifungal activity was evaluated against *Aspergillus* sp., *Fusarium* sp. and *Alternaria alternata*. Results showed a strong inhibitory effect of *Cl*EO on the growth of *A. niger* and *A. flavus* with inhibition zone diameters of 26 mm and MIC values of 0.625 and 0.312 mg/ml respectively (Table 3). Also, the *Cl*EO exhibited an antifungal activity against *Fusarium* sp. and *Aspergillus* sp., which are responsible for spoilage of many foods. The maximal inhibition zone diameters were 17–21 mm and MIC values ranged from 0.625 to 1.25 mg/ml (Table 3). The high antifungal activity may be attributed to the presence of the said chemical components in the essential oil. The antimicrobial activity of the essential oils of *C. limon* apparently related to its terpenes type components such as pinene, myrcene and limonene (Table 1), since there is a relationship between the chemical structures of the most abundant oils and their antimicrobial activities. Limonene has been shown to have strong antifungal properties [38]. Although the mechanism of action of terpenes is not fully understood, it is thought to involve membrane disruption by the lipophilic compounds [39]. The essential oils containing terpenes are also reported to possess antimicrobial activity [40], which are consistent with our present studies.

**Table 3:** Antifungal activity of *Cl*EO and determination of the Minimum Fungicidal Concentrations (MFCs) expressed in mg/ml

Fungal strains	Inhibition zones diameter (mm)^a^		MFC (mg/ml)
	EO^b^	Amphotericin B^c^	
*Aspergillus niger* (CTM 10099)	26 ± 0.9^b^	15 ± 0.9^a^	0.625 ± 0.4
*Aspergillus flavus* (food isolate)	26 ± 0.6^b^	10 ± 0.3^a^	0.312 ± 0.6
*Aspergillus nidulans* (food isolate)	20 ± 0.6^b^	0^a^	0.625 ± 0.5
*Aspergillus fumigatus* (food isolate)	18 ± 0.4^b^	0^a^	0.625 ± 0.7
*Fusarium graminearum* (ISPAVE 271)	20 ± 0.4^b^	14 ± 0.5^a^	0.625 ± 0.6
*Fusarium oxysporum* (CTM10402)*,*	18 ± 0.5^b^	14 ± 0.2^a^	0.625 ± 0.5
*Fusarium culmorum* (ISPAVE 21w)	21 ± 0.7^b^	12 ± 0.7^a^	0.312 ± 0.8
*Alternaria alternata* (CTM 10230)	17 ± 0.6^b^	14 ± 0.6^a^	1.25 ± 0.6

Values are given as mean ± SD of triplicate experiments

^a^Diameter of inhibition zones including diameter of disc 6 mm

^b^ *EO Lemon citrus* essential oil (50 μl/well)

^c^The used concentration of amphotericin B was 20 μg/well

Although the different compounds exhibited varying degrees of antifungal activity, β-caryophyllene and caryophyllene oxide were very fungitoxic against the studied Fusarium species [37]. Another minor monoterpene alcohol, linalool, is reported to have a wide range of antibacterial and antifungal activity [41]. Enatiomers of α-pinene, 2-β-pinene and limonene have a strong antibacterial activity [42]. These chemical components exert their toxic effects against studied microorganisms through the disruption of bacteria or fungal membrane integrity [43].

Moreover, the inhibitory activity of an essential oil is known to result from a complex interaction between its different constituents, which may produce additive, synergistic, or antagonistic effects, even for those present at low concentrations. Essential oils, which are odorous and volatile products of plant secondary metabolism, have wide applications in the food flavouring and preservation industries [33]. The results of this study suggest the possibility of using *Cl*EO or some of its components as natural food preservatives, because they possess strong antibacterial activity.

### Physicochemical analyses of treated meat samples

#### pH

The data presented in Table 4 indicate the pH evolution in raw minced beef meat treated with four different treatments. The initial pH recorded for the control and the treated samples was above 5.6. The pH value of (C) was found to raise from 5.66 ± 0.26 to 7.0 ± 0.24 at the end of storage period. Compared with control sample, no significant differences were found concerning pH value of T_1,_ T2 and T_3_ samples. Statistically, this difference of pH became significant (*P* < 0.05) from the sample T_4_. At the end of storage, pH values of raw minced meat were affected (*P* < 0.05) by the addition of preservatives. The pH rise (*P* < 0.05) reflects the degree of meat spoilage through protein breakdown for the production of free amino acids leading to the formation of NH_3_ and amines, compounds of alkaline reaction [44].

**Table 4:** Effect of *Cl*EO and their combination with BHT on pH and TBARS (mg of malonaldehyde equivalents per kg of sample (mg/kg) of raw minced meat beef during storage at 4 °C

Days of storage at 4 °C					
	0	2	4	8	10
pH					
C	5.66 ± 0.26^a^	5.82 ± 0.26^a^	6.37 ± 0.19^a^	6.85 ± 0.25^a^	7.0 ± 0.24^a^
T_1_	5.59 ± 0.21^a^	5.67 ± 0.24^a^	6.03 ± 0.18^a^	6.21 ± 0.18^b^	6.24 ± 0.26^b^
T_2_	5.57 ± 0.22^a^	5.62 ± 0.18^a^	6.1 ± 0.17^a^	6.17 ± 0.17^b^	6.19 ± 0.22^b^
T_3_	5.52 ± 0.22^a^	5.62 ± 0.14^a^	6 ± 0.15^a^	6.1 ± 0.18^b^	6.17 ± 0.23^b^
T_4_	5.57 ± 0.18^a^	5.62 ± 0.2^a^	5.9 ± 0.21^a^	6.05 ± 0.21^c^	6.10 ± 0.25^c^
TBARS					
C	0.2 ± 0.14^a^	0.88 ± 0.18^a^	1.57 ± 0.19^a^	2.31 ± 0.3^a^	3.96 ± 0.15^a^
T_1_	0.2 ± 0.08^a^	0.6 ± 0.12^b^	0.9 ± 0.13^b^	1.33 ± 0.3^a^	1.8 ± 0.15^b^
T_2_	0.18 ± 02^a^	0.4 ± 0.1^c^	0.75 ± 0.2^b^	0.83 ± 0.2^b^	1.7 ± 0.13^b^
T_3_	0.20 ± 0.14^a^	0.34 ± 0.12^c^	0.35 ± 0.16^c^	0.66 ± 0.21^c^	1.1 ± 0.11^c^
T_4_	0.19 + 0.19^a^	0.22 + 0.1^d^	0.28 + 0.3^c^	0.33 + 0.16^d^	0.55 ± 0.12^d^

±: Standard Error

^a-d^Averages with different letters in the same column, for each parameter, are different (*P* < 0.05); C (Control), T_1_ (EO: 0.06%), T_2_ (EO: 0.312%), T_3_ (BHT: 0.01%), T_4_ (EO 0.312% and BHT 0.01%)

#### TBARS

TBARS values represent the content of secondary lipid oxidation products, mainly aldehydes and carbonyls of hydrocarbons, which cause off-aromas in meat [45]. Over the storage period, TBARS values were higher in control samples (C) than treated samples (Table 4). The lowest TBARS values (0.55 ± 0.12 mg of malonaldhyde/kg) were achieved by treatment T4 after 10 days. It has been reported that an index of 2 was considered the limiting threshold for the acceptability of oxidised beef [46]. These results indicated that *Cl*EO/BHT (T_4_) exhibited higher antioxidant performance compared with *Cl*EO treatments (T_1_ and T_2_). However, antioxidant effects of BHT at 0.01% (T_3_) can not be ignored. Furthermore, those treated samples (T1, T2 and T3) were noted to remain below the detection limit (Table 4). The antioxidant performance of *Citrus* EO could be attributed to their phenolic contents found in leaves [4].

### Kill-time analysis: Effect of *Cl*EO on viable counts of *Listeria monocytogenes* ATCC 19117

**Fig. 3:**
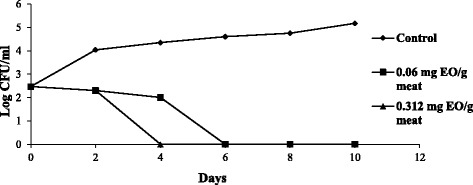
Time-related survival of *L. monocytogenes* at 4 °C following treatment with increasing concentrations of C*l*EO. Bacteria were supplemented in minced beef meat samples at 10^6^ CFU/g of meat. Values are the average of three individuals replicates

Controlling *Listeria monocytogenes* in food has become a major preoccupation in the food industry and storage. The purpose of this experiment was to determine the efficacy of *Cl*EO to repress the growth of *Listeria monocytogenes* ATCC 19117 in raw minced meat beef under storage at 4 °C. Therefore, the effect of *Cl*EO on *Listeria monocytogenes* ATCC 19117 was investigated to confirm its effect and clarify its action mechanism. The kinetics was performed using different concentrations of *Cl*EO (2 MIC and 3MIC). As shown in Fig. 3, at day 0, the number of *L. monocytogenes* was found to be the same for all meat samples (*P* > 0.05). Over the storage period at 4 °C, numbers of *L. monocytogenes* were higher in untreated samples than treated samples with 0.06 and 0.312 mg of *Cl*EO /g of raw minced meat beef: at a concentration of 2 and 3 MIC, growth of *L. monocytogenes* count was reduced by 2.5 log cycles after 4 and 6 days of storage at 4 °C respectively (Fig. 3).

Equally, the storage time had a significant effects (*P* < 0.05) on the growth of *L. monocytogenes* (Fig. 3). *Cl*EO at 0.06 and 0.312 mg have a significant effect (*P* < 0.05) on the growth of *L. monocytogenes*. The number of *L. monocytogenes* remains below a significant value (*P* < 0.05) till the end of the experiment (10 days). In fact, the addition of *Cl*EO at 2 and 3 MIC could substantially delay *Listeria monocytogenes* (*P* < 0.05) during raw minced meat beef storage at 4 °C.

## Conclusions

In the present work, we reported the phytochemical and biological properties of Tunisian essential oil from flowers of *Cirus limon*. The chemical composition of *Cl*EO revealed that limonene and β-Pinene were the main components. We also demonstrated, in vitro and in situ, the efficiency of *Cl*EO as a natural antioxidant and antimicrobial agent. In this regard, beef meat including *Cl*EO is an interesting target during refrigerated storage as contamination of beef meat by food spoilage and food-borne pathogens is considered one of the major problems to the progress of food industry.

Fractionation and characterization of *Cl*EO active compounds will be the future work to investigate; but also several other characterizations of *Citrus limon* will be worthwhile to contribute to a better valorization of this medicinal plant.

## Abbreviations

ATCC: American type culture collection
BHT: Butylated hydoxytoluene
CFU: Colony forming unit
*Cl*EO: *Citrus limon* essential oil
DMSO: Dimethylsulfoxide
DPPH: 2.2-diphenyl-1-picrylhydrazyl
IC_50_: Extract concentration providing 50% inhibition
MDA: malonaldehyde
MFCs: Minimum fungicidal concentrations
MH: Muller-Hinton broth
MICs: Minimum inhibitory concentrations
MTT: Thiazolyl blue tetrazolium bromide
PDA: Potatoes dextrose agar
SD: Standard deviation
TBARS: Thiobarbituric acid reactive substances
TCA: Trichloroacetic acid
